# Ocular Hornet Injury: A Case Report on Corneal Microperforation and Endophthalmitis

**DOI:** 10.7759/cureus.65515

**Published:** 2024-07-27

**Authors:** Muhammad Hafiz As-Shaarani Mohd Amin, Abdul-Hadi Rosli, Adzura Salam

**Affiliations:** 1 Ophthalmology, Kulliyyah of Medicine, International Islamic University Malaysia, Kuantan, MYS; 2 Ophthalmology, Kulliyyah of Medicine, International Islamic University Malaysia, Kuala Lumpur, MYS

**Keywords:** endophthalmitis, corneal microperforation, hornet stings, lesser banded hornet, vespa affinis indosinensis

## Abstract

The lesser-banded hornet (*Vespa affinis indosinensis*) is a prevalent species in tropical and subtropical regions of Asia, including Malaysia. Its stings can result in local reactions, severe anaphylactic shock, and even death. We report a rare case of corneal microperforation and endophthalmitis following an ocular hornet injury. A 76-year-old farmer was attacked by hornets and suffered multiple stings, including one to his right eye. He developed right eye pain, redness, and visual impairment. Ocular examination revealed right corneal haziness with a retained stinger. Attempts to remove the retained stinger were unsuccessful, as the stinger broke and stayed deep in the corneal tissue layer. Corneal microperforation occurred at the site of the retained stinger. Subsequently, he developed endophthalmitis despite extensive topical and systemic antibiotics. He also required a scleral patch procedure for sclerokeratouveitis. This report highlights the importance of quick and vigilant management to prevent severe complications and preserve vision after a hornet sting injury. Retained stingers pose unique challenges that require specialized interventions. There is a need for continuous research and awareness in the management of ocular hornet injuries, aiming to establish standardized treatment guidelines and improve patient outcomes.

## Introduction

The lesser-banded hornet (*Vespa affinis indosinensis*) is a common hornet in tropical and subtropical Asia, including Malaysia [1]. Hornet sting injuries have been reported as rare incidents, but their potential severity and complications make it important to understand the nature of such occurrences. Hornets, with their black and white bodies and yellow head strips, are known for protecting their nests fiercely and can become aggressive when they perceive any disturbance in their vicinity [2]. Disturbances to their nests can lead to multiple sting injuries, which can have unpredictable complications and even result in death [3]. These stings can cause allergic reactions in victims, ranging from local cutaneous manifestations to potentially life-threatening anaphylactic reactions [4,5]. Furthermore, hornet stings have been associated with a range of systemic and organ-specific complications, including myocardial infarction, multiple organ failure, myasthenia gravis, mastocytosis, and reversible optic neuropathy [6]. The incidence of ocular complications following hornet stings depends on factors such as the location and extent of stings, the presence of retained stingers, and individual patient characteristics. In this case report, we present a rare case of corneal microperforation and endophthalmitis following an ocular hornet injury. This article was previously presented as a meeting abstract at the International Virtual Medical Research Symposium 2023 on December 7 and 8, 2023.

## Case presentation

A 76-year-old male was attacked by a swarm of hornets on his palm oil plantation. He suffered multiple hornet stings over his face, bilateral hands, and right eye. At the primary health care facility, he received intravenous hydrocortisone, intramuscular pain relief, and intramuscular adrenaline to alleviate pain and address potential allergic reactions. Eventually, he complained of right eye pain, redness, gritty sensations, and reduced vision. He was referred to the ophthalmology department for further evaluation and management.

**Figure 1:**
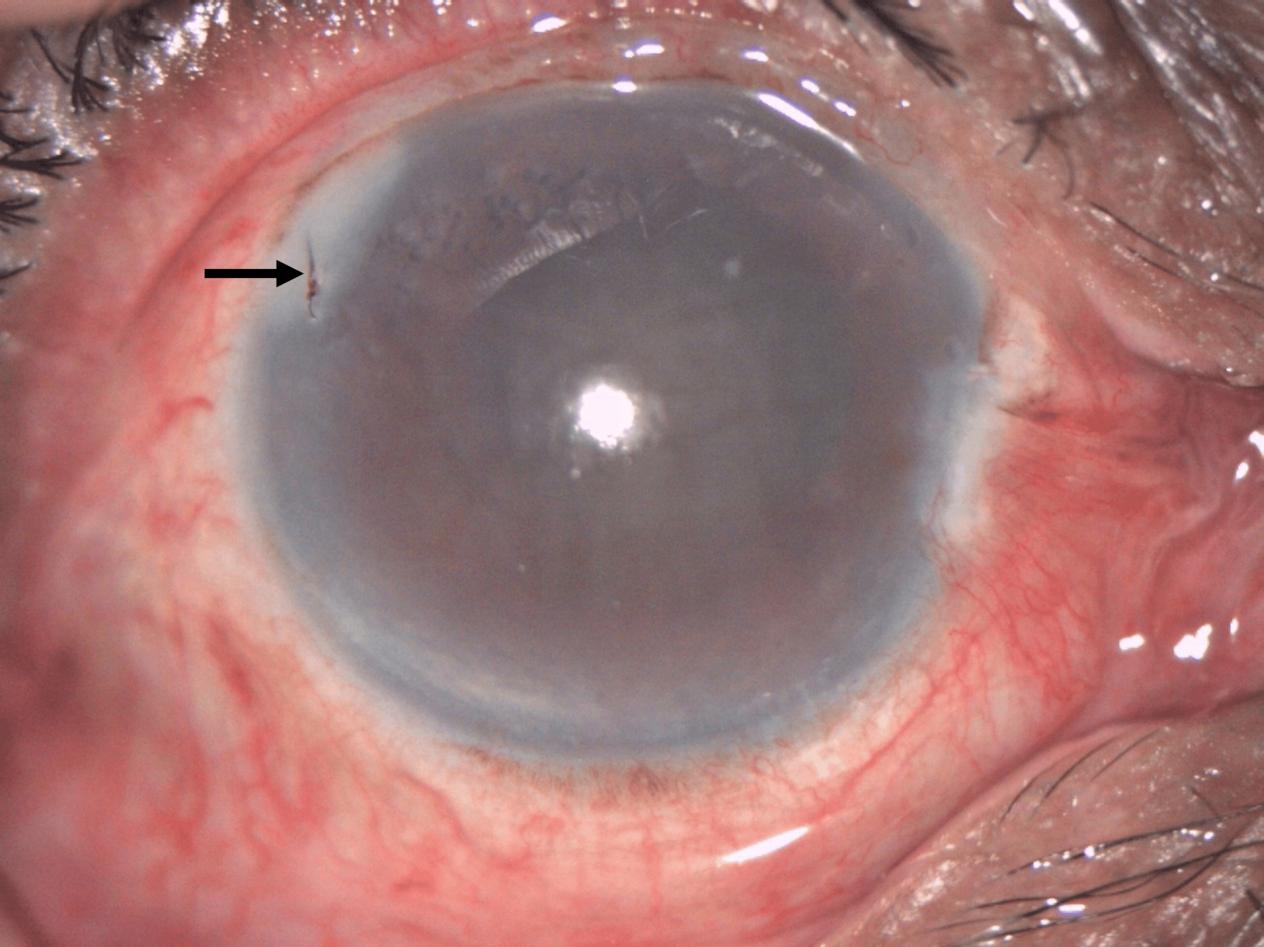
Anterior segment image of the right eye showing a retained hornet stinger (black arrow) at the peripheral 10 o’clock position with a generalized hazy cornea

Upon examination, the visual acuity in the right eye was 6/60 unaided, with no improvement on pinhole testing. The left eye had a visual acuity of 6/7.5 unaided. Intraocular pressure was within normal limits in both eyes. The anterior segment examination of the right eye showed generalized haziness in the cornea, with a retained stinger in the peripheral area at 10 o’clock (Figure 1). The anterior chamber was deep, with no signs of inflammation. Fundoscopic examination was limited due to corneal edema. The left eye, conversely, had a clear cornea with nuclear sclerosis grade 1.

Attempts to remove the retained stinger were unsuccessful as the stinger broke, thus halting the removal of the whole stinger. He was started on topical moxifloxacin 0.5%, topical ceftazidime 5%, topical dexamethasone 0.1%, and intravenous ciprofloxacin 400 mg twice daily. On day 2 of the injury, corneal microperforation occurred at the site of the retained stinger, which was followed by toileting and suturing to the affected area.

**Figure 2:**
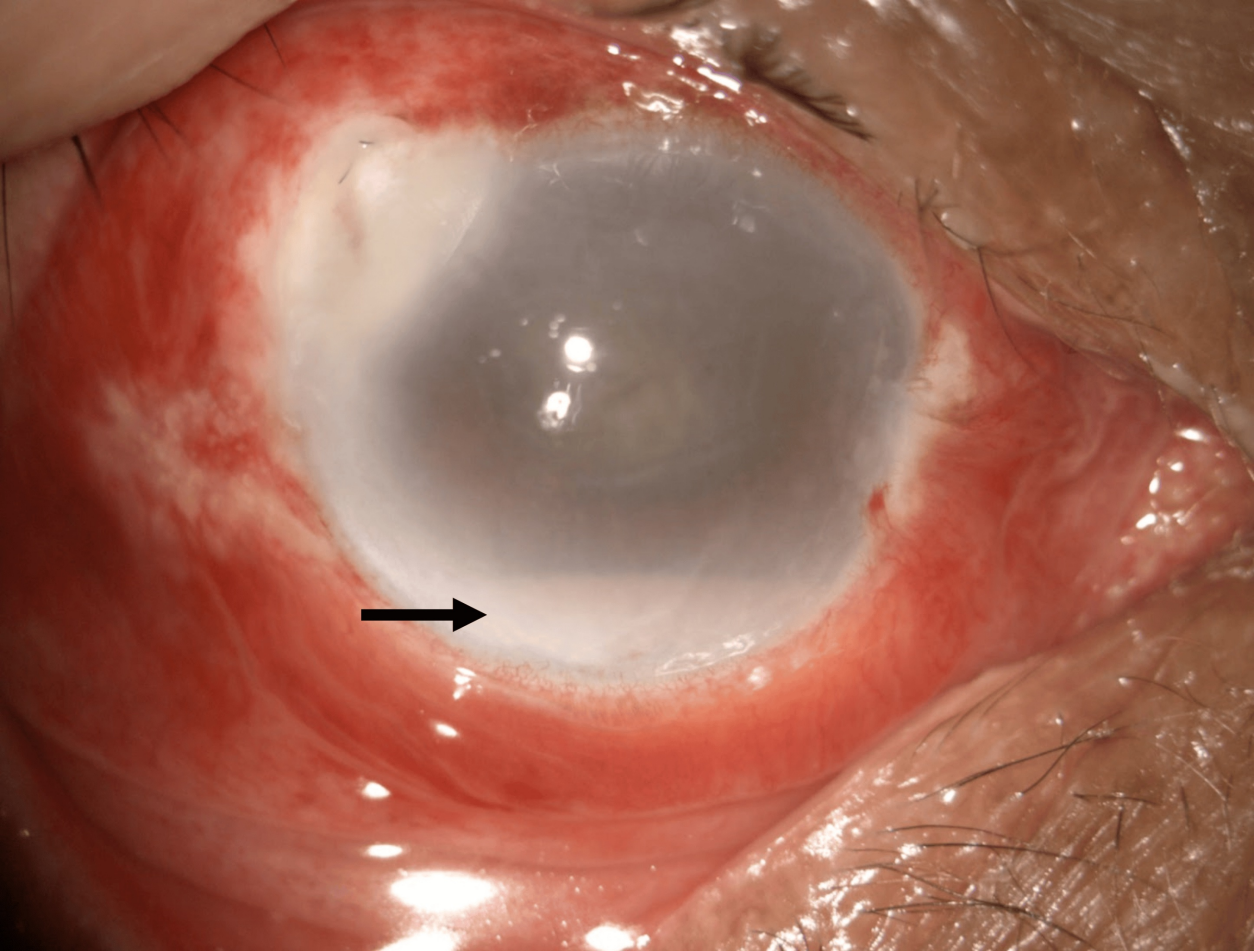
Right eye anterior segment photo showing hypopyon (black arrow)

**Figure 3:**
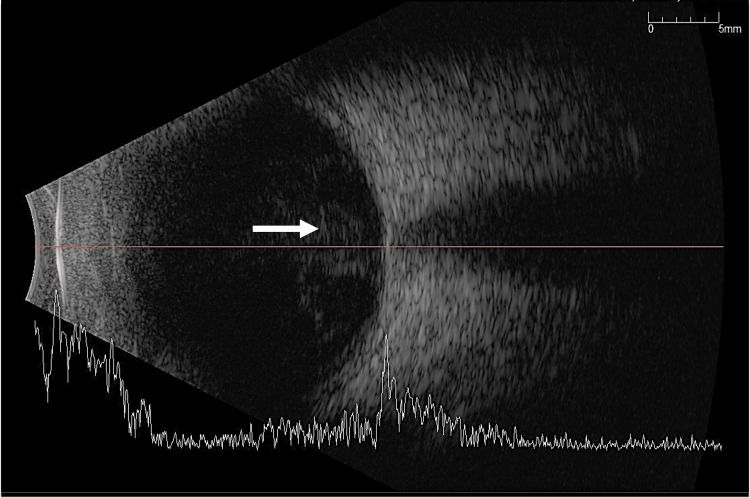
B-scan of the right eye showing aggregates of vitritis near the posterior pole (white arrow)

He developed endophthalmitis subsequently on day 3 of the injury. There was a presence of hypopyon (Figure 2), and a B-scan showed vitritis (Figure 3). We proceeded with anterior chamber washout; intravitreal vancomycin (1 mg in 0.1 ml), ceftazidime (2 mg in 0.1 ml), and amphotericin B (5 mcg in 0.1 ml) were given in the same setting. Intravitreal amphotericin B was given to prevent fungal infection. Trans pars plana vitrectomy was not performed due to a hazy cornea.

**Figure 4:**
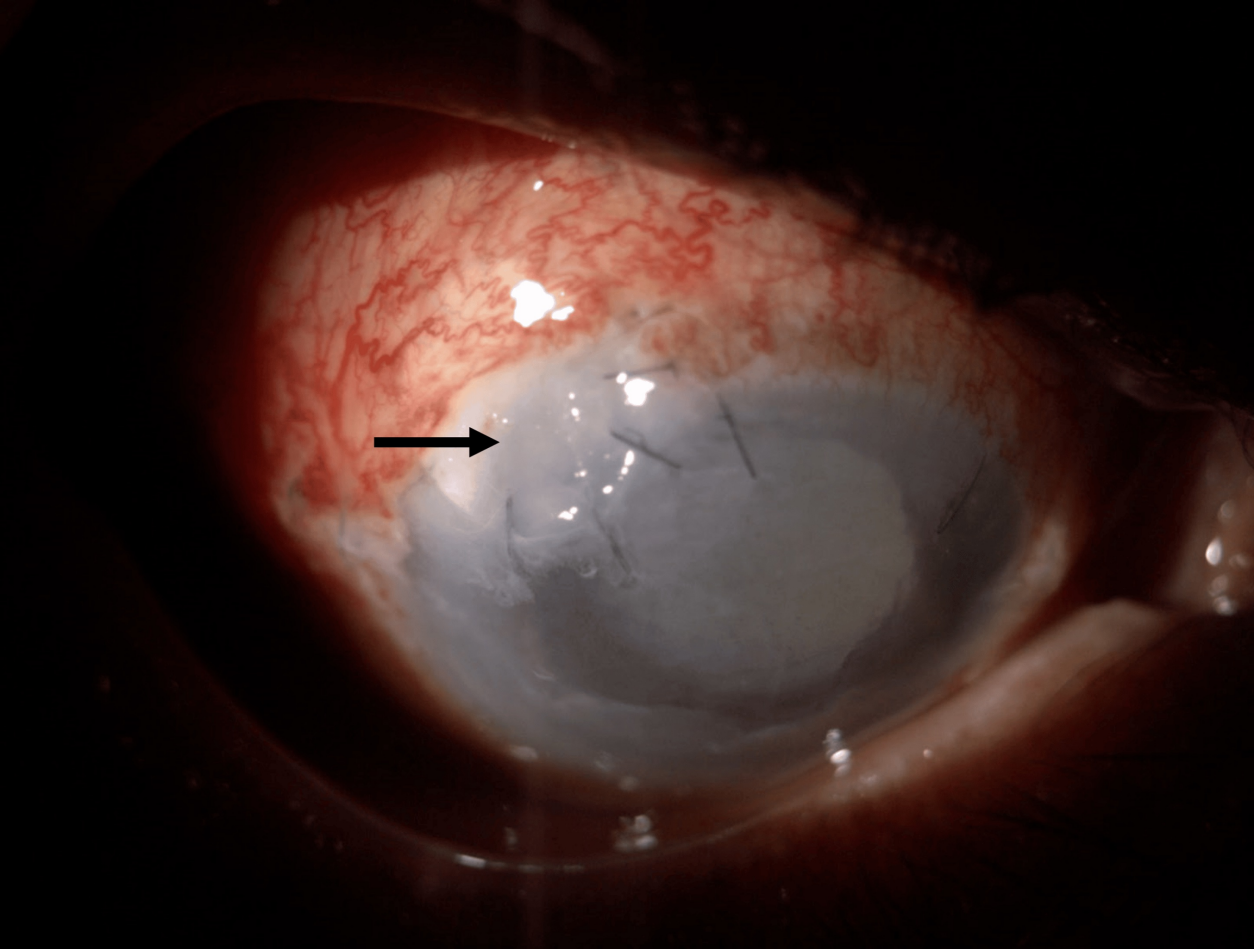
Postoperative image of the right eye following the scleral patch procedure due to microperforation (black arrow)

Eventually, he developed sclerokeratouveitis on day 11 of the injury, which required a scleral patch procedure (Figure 4). He was closely monitored during his hospitalization, which spanned approximately two weeks, in terms of any signs of infection recurrence, corneal healing, and intraocular pressure fluctuations. He was then discharged with topical moxifloxacin, topical dexamethasone, and artificial tears. Follow-up appointments were scheduled to assess the patient’s progress and address any potential post-treatment complications.

## Discussion

This case report highlights the severity of complications that can arise from ocular hornet injuries. While most individuals experience localized reactions to hornet stings, systemic reactions can occur, leading to severe complications such as corneal microperforation and endophthalmitis [4,7,8]. The ocular involvement in this case was particularly challenging due to the presence of the retained hornet stinger, which increased the risk of ongoing inflammation and infection. Ocular hornet injuries are relatively rare, and the management of such cases can be complex and requires a multidisciplinary approach involving ophthalmologists, infectious disease specialists, and other relevant healthcare professionals. To date, there are no standardized treatment guidelines for ocular hornet injuries, highlighting the need for further research and consensus among medical experts.

In light of this, early recognition of retained ocular foreign bodies following hornet stings is paramount to reduce the risk of severe complications. The occurrence of corneal microperforation in this case underscores the potential for severe ocular damage in ocular hornet injuries. The scleral patch procedure employed is a valuable technique for managing corneal microperforation and preventing devastating outcomes [9].

Additionally, the development of exogenous endophthalmitis is an alarming complication, warranting a closer look at the management of retained ocular foreign bodies. The retained hornet stinger served as a nidus for infection, propagating the inflammatory process and increasing the risk of vision-threatening complications. Anterior chamber washout and the use of intravitreal antibiotics serve as examples of targeted and localized therapy to combat developing endophthalmitis and reduce the microbial load intraocularly [10]. Although the treatment proved successful in this case, further research is needed to explore the optimal antimicrobial agents and dosing strategies for intravitreal injections in ocular hornet injuries.

## Conclusions

Immediate and vigilant management is crucial in cases of ocular hornet injuries to prevent serious complications and preserve visual function. The presence of a retained hornet stinger represents a unique challenge, necessitating specialized interventions to mitigate inflammation and infection risk. Healthcare providers should be prepared to manage potential complications associated with ocular hornet injuries, and close follow-up is essential to ensure successful outcomes.

This case report emphasizes the importance of continued research and awareness regarding the management of ocular hornet injuries to optimize patient care and outcomes. Further studies are warranted to establish standardized treatment guidelines and improve the management of ocular complications following hornet stings.
